# Aortic stenosis assessment from the 3-chamber cine: Ratio of balanced steady-state-free-precession (bSSFP) blood signal between the aorta and left ventricle predicts severity

**DOI:** 10.1016/j.jocmr.2023.100005

**Published:** 2024-01-09

**Authors:** Kavitha Vimalesvaran, Sameer Zaman, James P. Howard, Nikoo Aziminia, Marilena Giannoudi, Henry Procter, Marta Varela, Fatmatulzehra Uslu, Ben Ariff, Nick Linton, Eylem Levelt, Anil A. Bharath, Graham D. Cole

**Affiliations:** a A1 for Healthcare Centre for Doctoral Training, Imperial College London, SW7 2AZ, United Kingdom; b National Heart and Lung Institute, Imperial College London, SW7 2AZ, United Kingdom; c Imperial College Healthcare NHS Trust, London W12 0HS, United Kingdom; d Barts Heart Centre, Barts Health NHS Trust, West Smithfield, London EC1A 7BE, United Kingdom; e Multidisciplinary Cardiovascular Research Centre & Department of Biomedical Imaging Science, Leeds Institute of Cardiovascular and Metabolic Medicine, University of Leeds, Leeds LS2 9JT, United Kingdom; f Department of Electric-Electronic Engineering, Bursa Technical University, Bursa, Turkiye; g Department of Bioengineering, Imperial College London, SW7 2AZ, United Kingdom

**Keywords:** Aortic Valve, Aortic Stenosis, Left Ventricle, Balanced Steady-State Free Precession, Magnetic Field Strength, Valvular heart disease

## Abstract

**Background:**

Cardiovascular magnetic resonance (CMR) imaging is an important tool for evaluating the severity of aortic stenosis (AS), co-existing aortic disease, and concurrent myocardial abnormalities. Acquiring this additional information requires protocol adaptations and additional scanner time, but is not necessary for the majority of patients who do not have AS. We observed that the relative signal intensity of blood in the ascending aorta on a balanced steady state free precession (bSSFP) 3-chamber cine was often reduced in those with significant aortic stenosis. We investigated whether this effect could be quantified and used to predict AS severity in comparison to existing gold-standard measurements.

**Methods:**

Multi-centre, multi-vendor retrospective analysis of patients with AS undergoing CMR and transthoracic echocardiography (TTE). Blood signal intensity was measured in a ∼1 cm^2^ region of interest (ROI) in the aorta and left ventricle (LV) in the 3-chamber bSSFP cine. Because signal intensity varied across patients and scanner vendors, a ratio of the mean signal intensity in the aorta ROI to the LV ROI (Ao:LV) was used. This ratio was compared using Pearson correlations against TTE parameters of AS severity: aortic valve peak velocity, mean pressure gradient and the dimensionless index. The study also assessed whether field strength (1.5 T vs. 3 T) and patient characteristics (presence of bicuspid aortic valves (BAV), dilated aortic root and low flow states) altered this signal relationship.

**Results:**

314 patients (median age 69 [IQR 57–77], 64% male) who had undergone both CMR and TTE were studied; 84 had severe AS, 78 had moderate AS, 66 had mild AS and 86 without AS were studied as a comparator group. The median time between CMR and TTE was 12 weeks (IQR 4–26). The Ao:LV ratio at 1.5 T strongly correlated with peak velocity (r = −0.796, p = 0.001), peak gradient (r = −0.772, p = 0.001) and dimensionless index (r = 0.743, p = 0.001). An Ao:LV ratio of < 0.86 was 84% sensitive and 82% specific for detecting AS of any severity and a ratio of 0.58 was 83% sensitive and 92% specific for severe AS. The ability of Ao:LV ratio to predict AS severity remained for patients with bicuspid aortic valves, dilated aortic root or low indexed stroke volume. The relationship between Ao:LV ratio and AS severity was weaker at 3 T.

**Conclusions:**

The Ao:LV ratio, derived from bSSFP 3-chamber cine images, shows a good correlation with existing measures of AS severity. It demonstrates utility at 1.5 T and offers an easily calculable metric that can be used at the time of scanning or automated to identify on an adaptive basis which patients benefit from dedicated imaging to assess which patients should have additional sequences to assess AS.

## 1 Background

Aortic stenosis (AS) is a common valvular heart disease, with a prevalence rate of 3% among patients aged ≥ 75 years [1]. Transthoracic echocardiography (TTE) is the primary imaging modality for the assessment of AS [2], [3]. However, TTE can be limited by poor acoustic windows and is operator-dependent, particularly for quantifying disease severity [4]. Cardiovascular magnetic resonance (CMR) has emerged as a useful tool in assessing valvular heart disease severity [5], [6], [7] and is a reliable alternative to transoesophageal echocardiography (TEE) and cardiac catheterisation, whilst also providing additional information about concurrent myocardial and aortic abnormalities [8].

Cine CMR sequences provide most of the morphologic and functional information required for assessing valvular heart disease [5]. These sequences typically obtain 20–40 images over the cardiac cycle in a single plane through the heart. They are usually acquired within a single breath-hold using a balanced steady-state free-precession (bSSFP) sequence readout, which offers high signal to noise ratio and intrinsic contrast between blood and the surrounding cardiac structures [9], [10]. The imaging plane selection usually consists of longitudinal and perpendicular imaging planes through the valve of interest [10].

While phase-contrast cardiovascular magnetic resonance imaging (PC-CMR) can provide quantification of turbulent blood flow across affected valves, bSSFP cines not only show valve leaflet motion and function (e.g., demonstrating physiological leaflet excursion during valve opening in AS), but also depicts alterations in the normal blood flow pattern [6], [10]. Turbulent flow patterns across affected valves, resulting from acceleration or loss of flow homogeneity, manifests as a region of reduced signal intensity (signal void) [6] due to intravoxel spin dephasing [11], [12]. The degree of this signal loss is dependent on pulse sequence design and imaging parameters such as echo time (TE) [6], [13], [14], [15].

### 1.1 Ratio of Aortic to Left Ventricular bSSFP signal loss

In CMR imaging, variability in imaging parameters across scanner vendors, protocols and magnetic field strengths means that it is not reliable to use absolute values [16], [17]. We propose the ratio of aortic to left ventricular (LV) bSSFP signal (Ao:LV ratio) rather than absolute values to mitigate for this variability. The Ao:LV ratio can easily be obtained from the standard 3-chamber cine which is almost always acquired in routine clinical CMR protocols.

We hypothesized that the Ao:LV signal ratio could serve as a surrogate marker for AS in CMR. This could facilitate real-time decision-making (either by technologists/radiographers or on an automated basis) about the value of further dedicated aortic imaging on an individualised, case-specific basis. The objective was not to replace TTE/TEE but rather to offer a way of enhancing the diagnostic utility of CMR, particularly in those who were not suspected of aortic valve disease until the time of scanning.

The aims of our study were: [1] to quantify the aorta and LV blood signal ratio in bSSFP images; [2] to validate the Ao:LV signal ratio against the final grading of AS determined by the gold-standard TTE parameters and PC-CMR; and [3] to assess the applicability of this novel approach across different scanner (field strengths), and patient (anatomical and physiological) characteristics.

## 2 Methods

### 2.1 Patients

This was a multi-centre retrospective study from four hospitals which historically have used scanners from different vendors. We included adult patients (age ≥ 18 years) with a clinical diagnosis of AS based on echocardiographic findings. Patients underwent both echocardiography and CMR within 1 year of each other. We only excluded patients with incomplete imaging data (patients who did not have both CMR and echo modalities) or poor image quality unsuitable for measurements (see examples on Additional File 1). We also identified a control group of similar patients who had both investigations, but no history of aortic stenosis on either CMR or echocardiography. Some patients in the control group were selected to have a dilated aortic root to accurately assess the Ao:LV applicability in this characteristic. Demographic data including age and sex were extracted from electronic health records.

### 2.2 Cardiac MRI protocol

All patients underwent CMR across three institutions using 1.5 Tesla (Siemens Aera and Siemens Skyra; Siemens Healthineers, Signa Artist; GE Healthcare, or Ingenia; Philips Healthcare) or 3 Tesla (Prisma, Siemens Healthineers or Achieva, Philips Healthcare) scanners with a phased-array receiver coil. The imaging protocol included standard bSSFP cine in a 3-chamber view. Typical (but variable) 1.5 T scan parameters were: repetition time/echo time ∼ 36.5 ms/1.18 ms, slice thickness ∼ 8 mm, flip angle = 59°, pixel spacing row x column = 1.46 × 1.46 mm^2^, and matrix size = 240 × 196. 3 T scanners had a longer repetition time/echo time ∼ 45.78 ms/1.43 ms, thinner slice thickness ∼ 6 mm and smaller flip angle = 41°. PC-CMR measurements, velocity encoding (VENC) ranged from 150 to 500 cm/s and were acquired in a single slice perpendicular to the aorta at the level of the aortic root.

### 2.3 Image analysis

The bSSFP images were analysed by experienced CMR observers (KV, NA, MG, HP) and evaluated using dedicated cardiac software (CVI42, Circle Cardiovascular Imaging, Inc., Calgary, Canada). To ensure accuracy and consistency, observers followed predefined rules during image analysis. Observers manually measured a region of interest (ROI) of approximately 1 cm^2^ in the aorta and left ventricle to avoid contamination from surrounding tissues or structures. The observers followed a convention of placing the ROI in areas away from the tissue-blood boundary to reduce the risk of partial volume effects. Specifically, the ROI for the aortic blood signal was measured in the centre of the ascending aorta, approximately 1 cm above the aortic valve leaflets. The ROI for the LV blood signal was chosen to be in the centre of the LV cavity, at the mid-papillary muscle level, avoiding the papillary muscles or the myocardium.

**Fig. 1:**
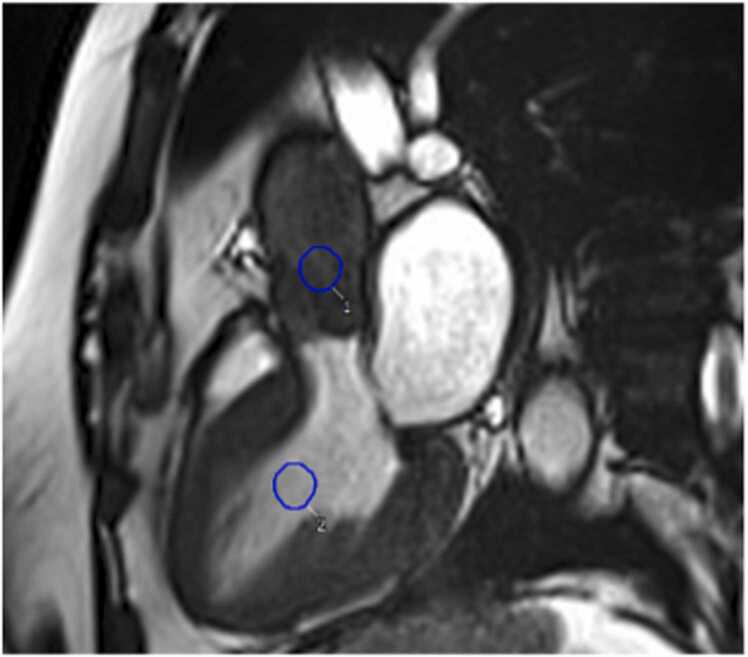
Example of a 3-chamber cine in a patient with aortic stenosis. A region of interest (ROI) in the aorta and left ventricle is manually measured in the end-systolic cardiac phase. The ratio of Ao:LV bSSFP blood signal in this patient: 67.5/186.2 = 0.36. Ao: aorta; LV: left ventricle; bSSFP: balanced steady state free precession.

Measurements were made in end-systole, visually identified as the frame with the smallest LV cavity size during the cardiac cycle after aortic valve closure. The end-systolic phase was chosen for this study because it represents the point in the cardiac cycle when the left ventricle stops contracting, and the aorta maintains relatively high pressures, maximising the haemodynamic differences between the LV and aorta. This phase also avoids contamination from turbulent AS jets during systole which can introduce inaccuracies in signal measurements (Additional File 2). The ratio of the aortic to LV blood signal was calculated by dividing the mean aortic blood signal by the mean LV blood signal in the ROIs (Fig. 1).

### 2.4 Echocardiography

All patients underwent transthoracic echocardiography using standard imaging protocols and techniques according to the guidelines of the British Society of Echocardiography (BSE) [4], [18]. The following parameters were measured: aortic valve area, peak aortic jet velocity, mean transvalvular gradient, peak transvalvular gradient and the dimensionless index. AS severity was determined using visual assessment and the peak aortic jet velocity and classified as mild (peak velocity < 3.0 m/s), moderate (peak velocity 3.0 – 4.0 m/s), or severe (peak velocity > 4.0 m/s). We took the overall clinical conclusion in the final report for categorising patients in the study.

### 2.5 Statistical analyses

Continuous variables are presented as mean ± standard deviation or median with interquartile range, depending on normality Categorical variables are presented as percentages and frequency. The correlation between Ao:LV ratio and AS severity was assessed using Pearson correlation coefficient. Receiver operating characteristic (ROC) curve analysis was performed to determine the optimal cut-off value of the bSSFP signal ratio for predicting severe AS. The diagnostic performance of the Ao:LV ratio was evaluated using sensitivity, specificity, and area under the ROC curve (AUC). Ao:LV ratios across varying degrees of AS and sub-groups were compared by the analysis of variance (ANOVA). Statistical significance was set at p < 0.05. All statistical analyses were performed using Numpy, Pandas and SciPy libraries in the Python3 programming language.

### 2.6 Inter-observer variability

Inter-observer variability was assessed in a subset of patients by two independent experienced CMR clinicians blinded to the clinical data and each other’s measurements. The readers independently performed ROI measurements using the same methodology as described above. The inter-observer variability was evaluated using the intraclass correlation coefficient (ICC) and Pearson’s correlation coefficient. Bland-Altman analysis was also performed to assess the agreement between the two readers.

### 2.7 Data integrity

CMR images were reviewed for quality and inclusion criteria by an experienced CMR clinician (GC) before inclusion in the study. KV and GC had full access to all the data in the study and take responsibility for its integrity and the data analysis.

## 3 Results

### 3.1 Baseline patient characteristics

A total of 314 patients were included in the study's final analysis, comprised of 228 with AS (84 severe, 78 moderate, 66 mild) and 86 controls without AS. The median age of the patients was 69 years (interquartile range [IQR]: 57 – 77), and 64% were male (Table 1). The median time between CMR and echocardiography was 12 weeks (IQR 4 – 26). Given the distinct imaging characteristics associated at 3 T, a separate analysis was conducted for this group.

**Table 1:** Baseline characteristics of 307 patients included in the study. Data are presented as mean + /- standard deviation of number (%).

	Control n = 86	Mild n = 66	Moderate n = 78	Severe n = 84
**Clinical Characteristics**				
Age, mean years± SD Male sex, n (%)	58 ± 15 48 (56%)	66 ± 10 41(62%)	68 ± 16 54 (69%)	72 ± 12 57 (68%)
**Echocardiography Characteristics**				
Peak Velocity (m/s) Dimensionless index Mean Pressure Gradient (mmHg) Peak Pressure Gradient (mmHg) AVA (cm^2^) Indexed AVA (cm^2^/m^2^) Aortic Regurgitation Grade ≥ 2 Mitral Regurgitation Grade ≥ 2 AS morphology Bicuspid, n (%) Tricuspid, n (%)	1.59 ± 0.61 0.65 ± 0.14 8 ± 7 11 ± 9 2.65 ± 0.93 1.32 ± 0.42 14 10 7 (8%) 79 (92%)	2.34 ± 0.63 0.47 ± 0.12 13 ± 7 24 ± 13 1.66 ± 0.42 0.87 ± 0.24 8 4 22 (33%) 44 (67%)	3.14 ± 0.68 0.33 ± 0.08 23 ± 11 42 ± 17 1.18 ± 0.32 0.65 ± 0.21 14 7 19 (24%) 59 (76%)	4.08 ± 0.89 0.26 ± 0.18 42 ± 19 72 ± 30 0.82 ± 0.23 0.45 ± 0.13 7 11 14 (17%) 70 (83%)
**CMR Characteristics**				
LV ejection fraction (%)	59 ± 14	62 ± 13	61 ± 15	60 ± 16
Indexed LV mass (g/m^2^)	75 ± 27	71 ± 20	76 ± 21	98 ± 34
Indexed LV EDV (ml/m^2^)	87 ± 43	78 ± 22	82 ± 32	83 ± 28
LV maximum wall thickness (mm)	10 ± 2	12 ± 3	13 ± 3	14 ± 3
Stroke volume index (ml/m^2^)	49 ± 17	46 ± 10	47 ± 14	47 ± 10
PC-CMR Peak Velocity	1.52 ± 0.54	2.18 ± 0.41	2.75 ± 0.64	2.88 ± 0.82
Maximum Ascending aorta size (mm)	35 ± 9	37 ± 6	36 ± 6	38 ± 5

AVA aortic valve area, AS aortic stenosis, CMR cardiac magnetic resonance imaging, LV left ventricular, EDV end-diastolic volume, SVi stroke volume index, PC phase contrast.

### 3.2 Ao:LV at different levels aortic stenosis severity

**Fig. 2:**
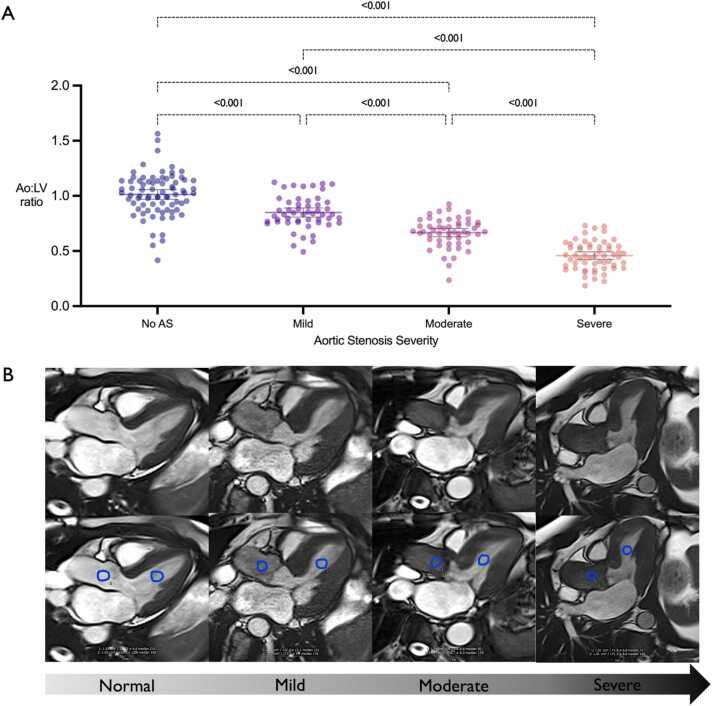
**A**. Dot plots for Ao:LV ratios according to aortic stenosis severity. Adjusted P values for Tukey’s multiple comparisons test. **B**. Example of four cases with varying degrees of aortic stenosis in the 3-chamber view with their corresponding Ao:LV ROI annotations. Ao:LV ratios are: Normal = 1.29, Mild AS = 0.70, Moderate AS = 0.61, and Severe AS = 0.41. ROI, region of interest; AS, aortic stenosis.

The ratio of aortic to LV blood signal was (mean ± standard deviation) 0.83 ± 0.14 in patients with mild AS, 0.67 ± 0.13 in patients with moderate AS, and 0.45 ± 0.12 in patients with severe AS. The difference in mean Ao:LV ratio between groups was statistically significant (p < 0.001). The Ao:LV ratio in the control group (1.01 ± 0.19) was significantly higher (p < 0.001) compared to patients with AS, regardless of severity (Fig. 2A).

### 3.3 Correlation between Ao:LV ratio and echocardiography and CMR parameters of aortic stenosis

**Fig. 3:**
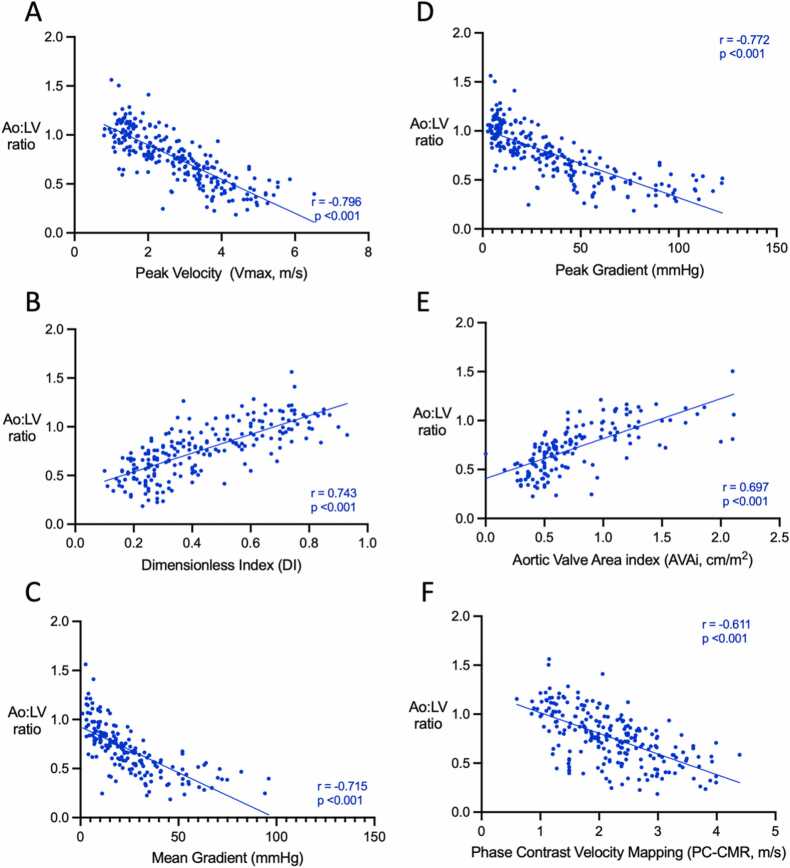
Results of comparisons between Ao:LV ratio, echocardiography, and phase contrast CMR. Scatterplots with Pearson correlation coefficients comparing Ao:LV and echo measures of peak velocity (A), dimensionless index (B), mean pressure gradient (C), peak pressure gradient (D), aortic valve area index (E) and peak velocity derived from PC-CMR (F). The trend suggested by Figs. 3C and 3D is of a gently decaying exponential, and not strictly linear; we have used a linear fit for consistency with the analysis for the remaining variables. PC, phase contrast; CMR, cardiovascular magnetic resonance; Ao: aorta; LV: left ventricle

Results are shown in Table 2), with the corresponding scatter plots in Fig. 3. The Ao:LV ratio had a strong negative correlation with echo peak velocity and peak gradient (r = −0.796, p < 0.0001; −0.772, p < 0.001, respectively), indicating a relationship between Ao:LV ratio and existing measurements of AS severity. Furthermore, the Ao:LV ratio also showed a strong positive correlation with the dimensionless index (r = 0.743, p < 0.001), suggesting that the ratio could be a valuable tool for assessing the haemodynamic significance of AS.

**Table 2:** Pearson correlation coefficients and p-values comparing parameters for 1.5 T magnetic field strength.

	Pearson’s r	p-value
TTE		
Peak Velocity (m/s)	-0.796	< 0.001
Dimensionless Index	+ 0.743	< 0.001
Mean Pressure Gradient (mmHg)	-0.715	< 0.001
Peak Pressure Gradient (mmHg)	-0.772	< 0.001
Aortic Valve Area (cm^2^)	+ 0.638	< 0.001
Aortic Valve Area Index (cm^2^/m^2^)	+ 0.697	< 0.001
CMR		
PC-CMR Peak Velocity (m/s)	-0.611	< 0.001

TTE transthoracic echocardiography, CMR cardiac magnetic resonance.

### 3.4 Diagnostic performance of Ao:LV Ratio – 1.5 T

**Fig. 4:**
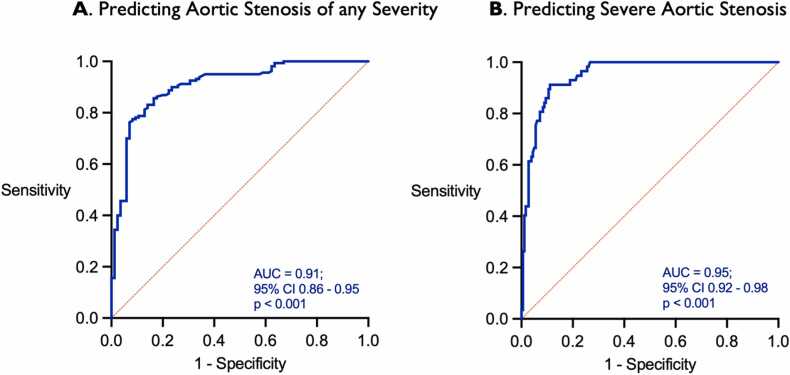
A. ROC curve showing the diagnostic accuracy for Ao:LV ratio in predicting AS of any severity. B. ROC curve showing the diagnostic accuracy for Ao:LV ratio in predicting severe AS. AS, aortic stenosis. ROC: receiver operating characteristic curve; Ao: aorta; LV: left ventricle.

Receiver operating characteristic (ROC) analysis was performed to evaluate the diagnostic performance of the Ao:LV ratio for the detection of AS (Fig. 4). The area under the ROC curve (AUC) was 0.91 (95 CI: 0.86 to 0.95), indicating excellent diagnostic performance. An Ao:LV cut-off value of 0.86 (i.e., a signal intensity in the aorta was less than 86% of that in the LV) had a sensitivity of 84% and specificity of 82% for identifying AS of any severity. An Ao:LV cut-off value of 0.58 (i.e., signal intensity in the aorta was less than 58% that in the LV had a sensitivity of 83% and a specificity of 92% for identifying severe AS.

### 3.5 Impact of Bicuspid Aortic valves on Ao:LV ratio

**Fig. 5:**
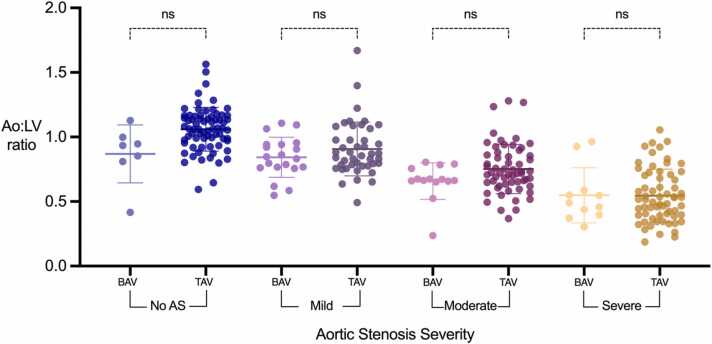
**:** Dot plots for Ao:LV ratios according to aortic stenosis severity and valve morphology. BAV, bicuspid aortic valve; TAV, trileaflet aortic valve; AS: aortic stenosis; Ao: aorta; LV: left ventricle.

Two hundred and fifty-two patients (80%) had a tricuspid aortic valve, and 62 (20%) had a bicuspid aortic valve. We considered whether the correlation was present for both bicuspid aortic valve (BAV) and trileaflet aortic valve (TAV) patients. Although the mean values for Ao:LV ratio in patients with BAV appear to be lower than those with TAV (Table 3) the difference was not statistically significant, as shown in Fig. 5. While the Ao:LV ratio significantly differs between severity groups, it does not serve as a useful differentiating metric between BAV and TAV within the context of AS severity.

**Table 3:** Comparison of mean ratio across different levels of aortic stenosis severity for tricuspid and bicuspid aortic valves.

	Control	Mild	Moderate	Severe
Mean Ao:LV ratio				
**Tricuspid**	1.06	0.91	0.75	0.55
**Bicuspid**	0.87	0.85	0.72	0.57

aorta:Ao:left, ventricle:LV.

### 3.6 Impact of low stroke volume on Ao:LV ratio

**Fig. 6:**
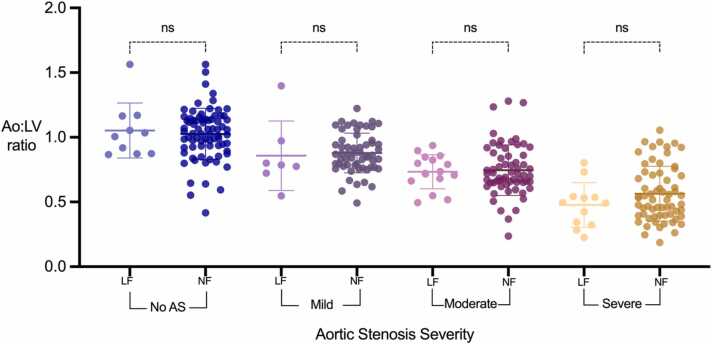
Dot plots for Ao:LV ratios according to aortic stenosis severity and differentiated into low (<35 ml/m2) and normal (>35 ml/m2) stroke volume index categories. LF, low flow; NF, normal flow; Ao: aorta; LV: left ventricle

There were 46 patients with a low flow state (stroke volume index ≤ 35 ml/m^2^); 10 in the control group, 8 in the mild group, 16 in the moderate group and 12 in the severe group. There was no significant difference in Ao:LV ratio between patients with low and normal stroke volume across all categories of AS severity (Fig. 6). No correlation was observed between Ao:LV ratio and the stroke volume index (Additional File 3).

### 3.7 Impact of a dilated aortic root on Ao:LV ratio

**Fig. 7:**
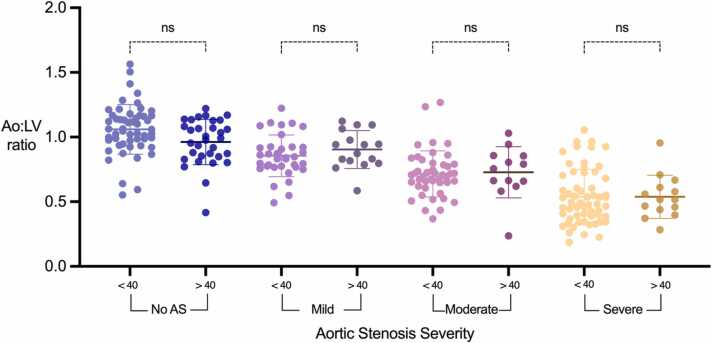
Dot plots for Ao:LV ratios according to aortic stenosis severity and differentiated into non-dilated (<40 mm) or dilated (>40 mm) aortic root dimensions. Ao: aorta; LV: left ventricle; AS: aortic stenosis.

There were 75 patients with a dilated aortic root (>40 mm at the annulus, sinuses of Valsalva or sinotubular junction): 32 in the control group, 16 in the mild group, 13 in the moderate group, and 14 in the severe group. There was no significant difference in Ao:LV ratio between patients with dilated and non-dilated aortic root size across all degrees of AS severity (Fig. 7), suggesting that aortic root dilatation does not have a notable impact on the Ao:LV ratio.

### 3.8 Impact of 3 T field strength on Ao:LV at different levels of aortic stenosis severity

**Fig. 8:**
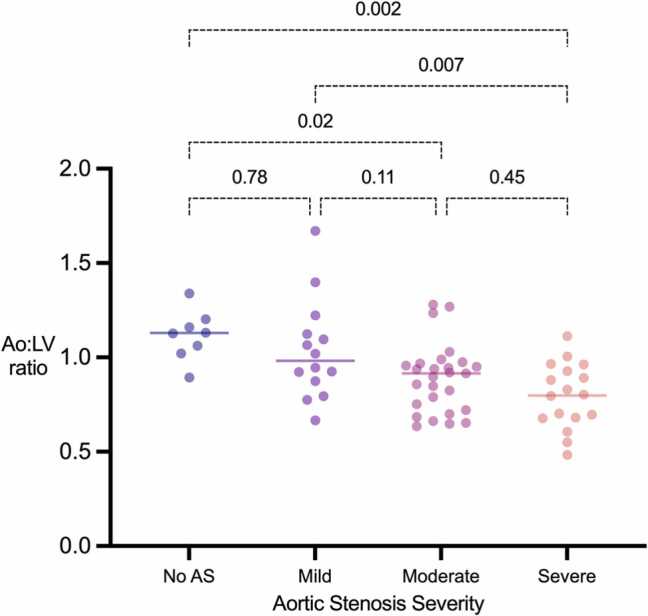
Dot plots for Ao:LV ratios according to aortic stenosis severity at 3 Tesla magnetic field strength. Adjusted P values for Tukey’s multiple comparisons test. Ao: aorta; LV: left ventricle; AS: aortic stenosis

Upon reviewing the 3 T images (n = 67), it was observed that the signal loss across degrees of AS was not as consistent as seen with 1.5 T data. This prompted a separate, focused analysis to understand the reliability and applicability of the Ao:LV ratio at 3 T. Tukey’s multiple comparisons test indicated less reliable differentiation between various AS severity levels at 3 T (Fig. 8) when compared to 1.5 T. There was a significant difference in the Ao:LV ratio between the control vs. severe and mild vs. severe groups (p = 0.002, p = 0.007 respectively). This suggests that while the method may not be uniformly reliable across all severities of AS at 3 T, it could potentially be useful in differentiating severe cases from those without AS and mild AS.

### 3.9 Intra and inter-observer variability

Inter-observer variability was quantified by blinded dual analysis 46 studies (15% of the overall sample) by two experienced CMR clinicians. Reader 1 analysed the images once, while Reader 2 analysed randomly selected images twice, with a time interval of two weeks between the two readings. There was good agreement between readers measuring the aorta and LV ROIs, and the same clinician measuring the Ao:LV twice. (Inter-observer Pearson’s correlation coefficient = 0.914 (p < 0.001); intra-observer Pearson’s coefficient = 0.953 (p < 0.001)).

## 4 Discussion

In this study we demonstrate a novel method, the Ao:LV ratio to approximating the severity of AS using the ratio of aortic to LV bSSFP blood signal in a single 3-chamber view. The Ao:LV ratio showed a strong correlation with peak aortic jet velocity, mean aortic gradient and the dimensionless index, which are considered gold standard echocardiographic measurements for assessing AS severity.

We assessed the influence of anatomical and physiological variables such as bicuspid aortic valves, dilated aortic root, and low stroke volume on the Ao:LV ratio. No significant different was observed in the Ao:LV ratio values when comparing BAV to TAV. Dilated aortic root and low stroke volume did not exhibit a significant impact on the Ao:LV ratio, further supporting its robustness for approximating AS severity without influence from anatomical variations or low flow states.

### 4.1 Correlation of Ao:LV ratio with PC-CMR

While the Ao:LV ratio shows significant correlation with standard TTE parameters, it exhibits significant yet comparatively weaker correlation with the peak velocity derived from PC-CMR (r = −0.611 compared to r = −0.796 in TTE derived peak velocity). This attenuated correlation may be attributable to several factors associated with the methodology of PC-CMR acquisition. Specifically, inconsistencies in selection imaging planes [19], [20], [21], could introduce variability in blood flow quantification and variations in VENC settings could affect the precision of flow measurements. The Ao:LV ratio could potentially be used as an initial estimator for determining the appropriate VENC settings.

### 4.2 1.5 T vs. 3 T magnetic field strength

We show that while the Ao:LV ratio is robust and clinically relevant at 1.5 T, it is less reliable at 3 T. The effectiveness of the Ao:LV ratio at 1.5 T is influenced by several factors. Firstly, 1.5 T bSSFP sequences exhibit lower sensitivity to off resonance effects compared to 3 T, leading to more reliable imaging [12], [22], [23]. Whilst this effect could be small, the need for meticulous shimming at 3 T could further complicate the clinical utility for the Ao:LV ratio [24]. Secondly, 1.5 T systems are currently the most widely utilised for CMR [22]. Their performance characteristics, imaging protocols, and sequence parameters are well-optimised and standardised that reduce variability in image quality, supporting the potential for broad application of the Ao:LV ratio.

Spin dephasing is an intrinsic factor that contributes to signal loss in CMR. In the context of AS, the increased turbulence and velocity of blood flow across the stenotic aortic valve can lead to significant spin dephasing due to interactions with magnetic field gradients. This seems to be reflected more consistently in the aorta and LV blood at 1.5 T. These changes in signal intensity are reflected in the Ao:LV ratio and could explain its ability to reflect the severity of AS accurately. As the severity of AS increases, the difference in signal intensity between the aortic and LV blood pool becomes more significant, leading to a lower Ao:LV ratio.

The ease and feasibility of measuring the Ao:LV ratio on routine CMR imaging is an important advantage of this method. The ratio can be easily calculated using standard software and does not require additional time or specialised imaging sequences. This means it could be incorporated into the standard CMR protocol or used as a reporting tool with minimal increase in time or cost. In addition, the study utilised scans from multiple different scanner vendors and centres, which demonstrates potential generalisability of the Ao:LV ratio method across different hardware.

Furthermore, the ability to measure this ratio using only routine sequences makes it a practical and accessible tool for clinicians to assess AS severity. Moreover, with the potential for automation of this measurement using machine learning algorithms, the Ao:LV ratio could potentially be obtained in real-time during the scanning process, allowing for immediate assessment of AS severity and facilitating prompt clinical decision-making and individualised sequence selection.

In summary, while specialised CMR imaging sequences have their place in the evaluation of AS and other cardiac conditions, the simplicity and efficiency of the Ao:LV ratio makes it a valuable tool for the initial diagnosis and assessment of severity in patients with suspected or known AS at 1.5 T. The ratio can potentially serve as a rapid triaging tool for deciding the necessity of specialised aortic valve sequences, predominantly for 1.5 T scanners until further validation is conducted for 3 T.

## 5 Limitations

Despite being a multi-centre, multi-vendor study, the study population was relatively small, particularly with respect to 3 T data. The evaluation of 3 T performance revealed that the correlation at this field strength was less secure and robust compared to 1.5 T despite promising trends at 3 T, notably between control and severe AS cases. Further research is essential to understand these nuances fully and to improve the robustness and applicability of the Ao:LV ratio across different magnetic strengths. Second, the non-significant impact of anatomical and physiological variables suggests that the ratio may not be an all-encompassing metric for assessing AS severity across these different conditions. Third, we did not assess the temporal reproducibility of the Ao:LV, which may be an important consideration in monitoring disease progression.

## 6 Conclusions

In conclusion, our study found that the novel Ao:LV ratio is a reliable marker for assessing the severity of AS and has potential clinical utility, particularly on 1.5 T systems. Its ease of measurement on routine CMR cine imaging and lack of need for additional time or specialised imaging sequences make it an attractive option for clinical use. Future studies are needed to validate our findings and further evaluate the reproducibility and clinical utility of the Ao:LV ratio. The potential for automation of this measurement, in the simplest case, by automatic placement of the regions, also holds promise for real-time assessment during the scanning process, which could further enhance the clinical utility of the Ao:LV ratio.

## Ethics approval and consent to participate

Ethical approval was gained from the Health Regulatory Agency for the Imperial, Barts and Leeds CMR Datasets; Integrated Research Application System ID 243023, 294495 and 252633 respectively.

## Consent for publication

All authors have approved the final manuscript and give consent for publication.

## Authors’ contributions

The study was conceived and designed by KV and GDC. Data curation was performed by KV. Manual annotations were performed by KV, SZ, NA, MG and HP. Data analysis was performed by KV and GDC. Data interpretation was performed by KV, JPH, MV, AAB and GDC. The manuscript was prepared KV, AAB and GDC. All co-authors reviewed and approved the manuscript prior to submission.

## Funding

KV is funded by the UK research and Innovation [10.13039/100014013UKRI Centre for Doctoral Training in AI for Healthcare grant number EP/S023283/1]. JH is funded by the 10.13039/501100000274British Heart Foundation [FS/ICRF/22/26039]. GDC is supported by the National Institute of Health Research (10.13039/100006662NIHR) Imperial Biomedical Research Centre (10.13039/100014461BRC).

## CRediT authorship contribution statement

**Varela Marta:** Investigation, Writing – review & editing. **Cole Graham D:** Conceptualization, Formal analysis, Investigation, Methodology, Supervision, Writing – review & editing. **Bharath Anil A:** Conceptualization, Data curation, Formal analysis, Investigation, Supervision, Writing – review & editing. **Levelt Eylem:** Data curation, Writing – review & editing. **Linton Nick:** Investigation, Methodology. **Zaman Sameer:** Data curation, Writing – review & editing. **Ariff Ben:** Investigation. **Howard James P:** Methodology, Supervision, Writing – review & editing. **Uslu Fatmatulzehra:** Writing – review & editing. **Aziminia Nikoo:** Data curation. **Giannoudi Marilena:** Data curation. **Procter Henry:** Data curation. **Vimalesvaran Kavitha:** Conceptualization, Data curation, Formal analysis, Investigation, Methodology, Project administration, Validation, Writing – original draft, Writing – review & editing.

## Declaration of Competing Interest

The authors declare that they have no known competing financial interests or personal relationships that could have appeared to influence the work reported in this paper.

## Data Availability

The datasets used and analysed during the current study are available from the corresponding author on reasonable request.
