# Supplementary material for: Aortic stenosis assessment from the 3-chamber cine: Ratio of balanced steady-state-free-precession (bSSFP) blood signal between the aorta and left ventricle predicts severity
Source: J Cardiovasc Magn Reson. 2024 Jan 9;26(1):100005. doi: 10.1016/j.jocmr.2023.100005 (PMC11211219; doi:10.1016/j.jocmr.2023.100005)
Supplement: Supplementary file 1 — Supplementary material [file mmc1.docx]

# Supplementary Information


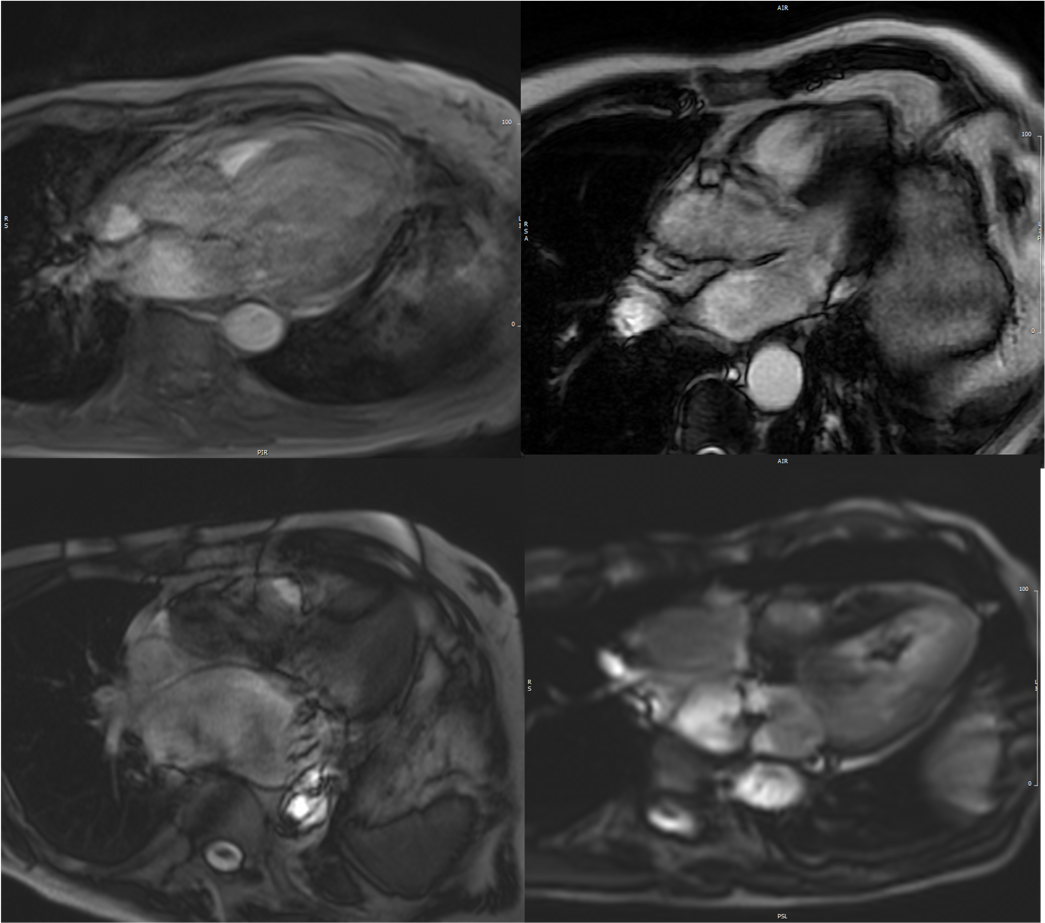
Additional File 1:

Additional File 1:Examples of excluded images due to poor image quality.

Additional File 2:

Additional File 2: Sequential cine frames captured on a bSSFP 3 chamber cine view demonstrating the Ao:LV ratio variability in a patient with severe aortic stenosis. Each frame is annotated with the calculated Ao:LV ratio. The frame highlights with a red box represents end-systole and exhibits the lowest Ao:LV ratio, consistent with the severity of aortic stenosis. The presence of turbulent jets within the ascending aorta during ventricular ejection contributes to the difficulty in accurately measuring the signal loss in this phase.


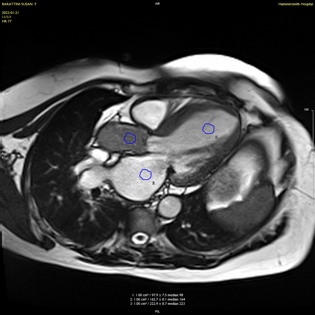

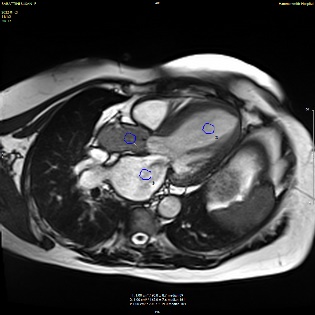

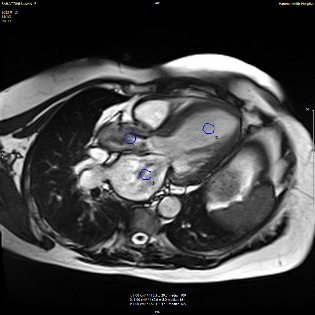

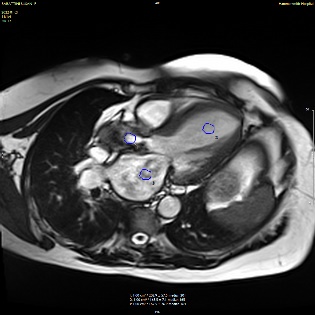

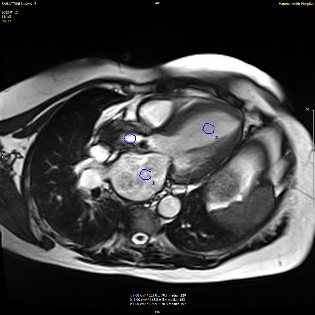

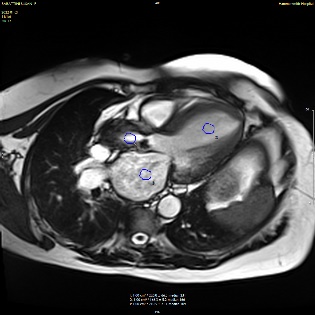

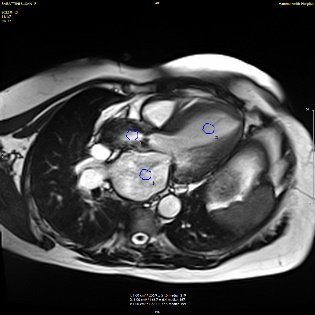

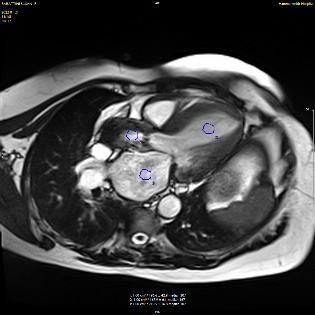

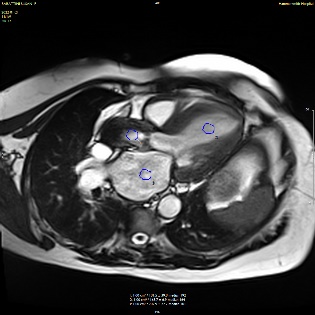

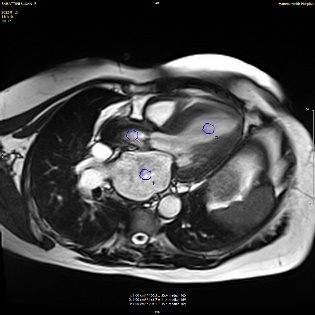

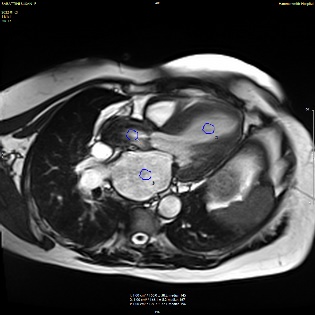

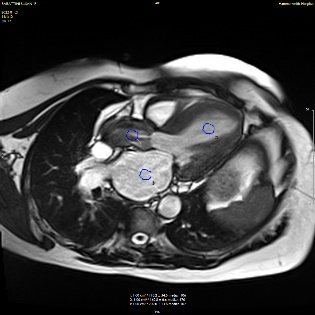

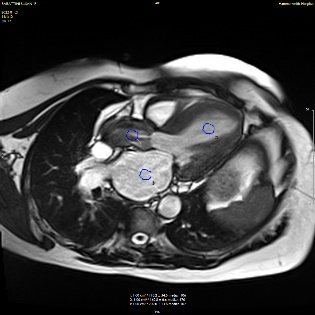

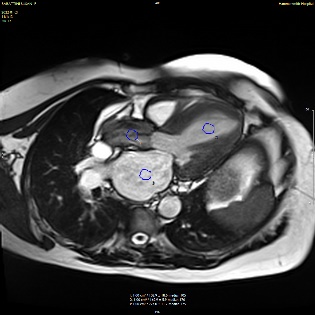

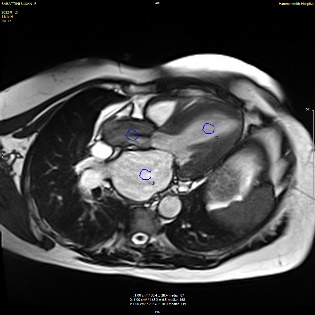

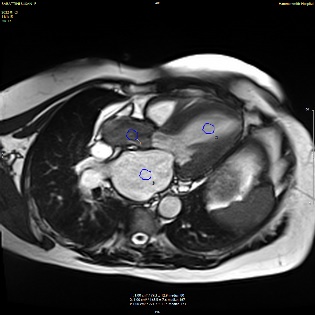

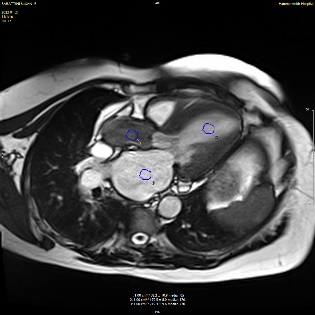

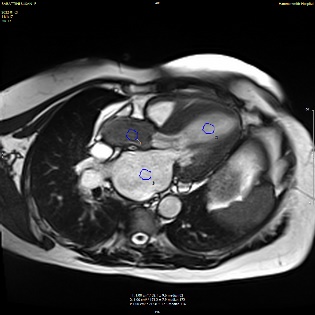

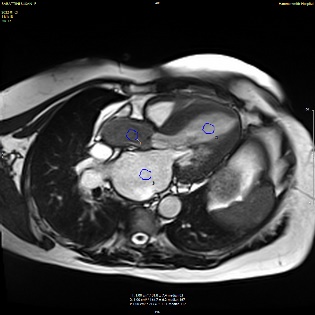

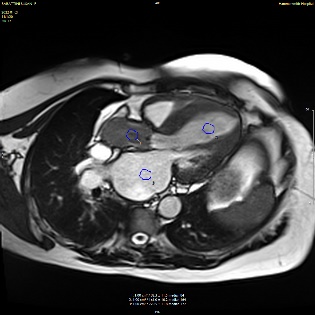

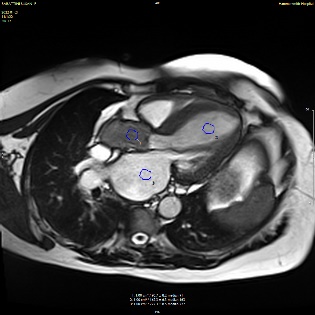

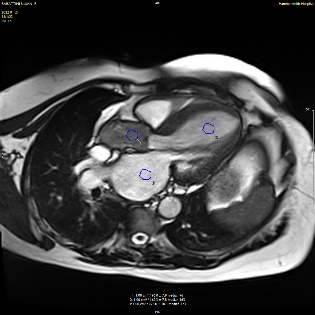

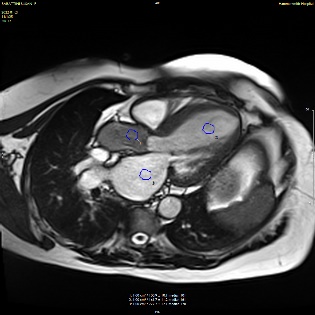

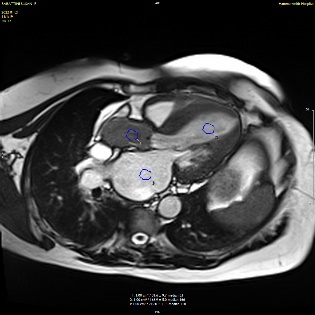

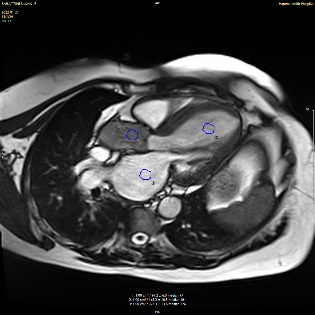


Ao:LV = 0.58

Ao:LV = 0.56

Ao:LV = 0.71

Ao:LV = 1.23

Ao:LV = 1.35

Ao:LV = 1.33

Ao:LV = 0.93

Ao:LV = 0.47

Ao:LV = 0.51

Ao:LV = 1.24

Ao:LV = 0.65

Ao:LV = 0.48

Ao:LV = 0.56

Ao:LV = 1.17

Ao:LV = 0.65

Ao:LV = 0.48

Ao:LV = 0.59

Ao:LV = 0.60

Ao:LV = 0.50

Ao:LV = 0.60

Ao:LV = 1.09

Ao:LV = 1.00

Ao:LV = 0.53

Ao:LV = 0.49

Ao:LV = 0.62

Additional File 3:

Additional File 3: Scatter plot of stroke volume index vs. Ao:LV ratio. The Pearson correlation coefficient (r) is 0.034, with p-value of 0.69, indicating no statistical significance in t
